# Assessment of lifestyle, blood pressure, and cholesterol in pharmaceutical industry professionals

**DOI:** 10.47626/1679-4435-2023-1070

**Published:** 2023-11-24

**Authors:** Cristiane Cremiude Ribeiro-Fernandes, Lara Lopes Faco, Danilo Costa Geraldes, Vivienne Carduz Castilho, Jairo Lins Borges, Manoel Patrocinio de Moraes Neto

**Affiliations:** 1 Gerência Ciências Médicas, Libbs Farmacêutica Ltda., São Paulo, SP, Brazil; 2 Departamento de Marketing, Libbs Farmacêutica Ltda., São Paulo, SP, Brazil; 3 Coordenação de Medicina do Trabalho, Libbs Farmacêutica Ltda., São Paulo, SP, Brazil

**Keywords:** occupational medicine, cardiovascular diseases, pharmaceutical industry, healthy lifestyle, medicina do trabalho, doenças cardiovasculares, indústria farmacêutica, estilo de vida saudável

## Abstract

**Introduction:**

Cardiovascular diseases are the leading cause of death worldwide.

**Objectives:**

To elucidate the lifestyle of in pharmaceutical company professionals, evaluating cardiovascular risk factors.

**Methods:**

This is an observational, longitudinal, and prospective study conducted with 1,875 individuals of both sexes. In addition to a questionnaire to identify participants’ lifestyle, calculation of body mass index, blood pressure measurement, and collection of blood samples to measure serum total cholesterol and glycated hemoglobin were performed.

**Results:**

83% of respondents had never smoked; 48.1% did not perform regular physical activity, and women tended to perform less physical activity than men; 57.6% consumed less than two servings of fruits or vegetables per day; 63.8% consumed fish less than once per week; 51.6% consumed less than one glass of sugary drinks per day, with women consuming fewer sugary drinks than men. Most participants had a body mass index from 25 to 29.9 m/kg^2^ or from 18.5 to 24.9 m/kg^2^ (43.6%), total cholesterol levels below 200 mg/dL (75.1%), glycated hemoglobin below 5.7% (86.0%), systolic blood pressure from 120 to 139 mmHg (47.6%), and diastolic blood pressure below 80 mmHg (56.1%).

**Conclusions:**

The data obtained in this study are consistent with those from the literature, demonstrating that it possible to improve habits such as smoking, diet, and physical activity.

## Abstract (pt)

**Introdução:**

As doenças cardiovasculares representam a maior causa de morte em todo o mundo.

**Objetivos:**

Elucidar o estilo de vida de profissionais de uma indústria farmacêutica, avaliando os fatores de risco cardiovascular.

**Métodos:**

Tratou-se de um estudo observacional, longitudinal e prospectivo, realizado com 1.875 indivíduos de ambos os sexos. Além de questionário para identificar o estilo de vida, foram realizados cálculo do índice de massa corporal, aferição da pressão arterial e coleta de amostra de sangue para dosagem de colesterol total sérico e hemoglobina glicada.

**Resultados:**

83% nunca tinham fumado; 48,1% não faziam atividade física regularmente e mulheres tendiam a realizar menos atividades físicas do que homens; 57,6% consumiam menos de duas porções de frutas ou verduras por dia; 63,8% consumiam peixe menos de uma vez por semana; 51,6% consumiam menos de um copo por dia de bebidas com açúcar, sendo que as mulheres consumiam menos bebidas açucaradas do que homens. A maioria dos participantes apresentou índice de massa corporal entre 25 e 29,9 m/kg^2^ ou entre 18,5 e 24,9 m/kg^2^ (43,6%), colesterol total abaixo de 200 mg/dL (75,1%), hemoglobina glicada abaixo de 5,7% (86,0%), pressão arterial sistólica entre 120-139 mmHg (47,6%), e pressão arterial diastólica menor que 80 mmHg (56,1%).

**Conclusões:**

Os dados são condizentes com informações de literatura, demonstrando que é possível melhorar hábitos como tabagismo, alimentação e prática de atividade física regularmente.

## INTRODUCTION

Cardiovascular diseases (CVDs) are the leading cause of death worldwide, especially in developing countries.^1^ Nearly 3/4 of deaths from CVDs occur in lowand medium-income countries like Brazil,^2^ possibly because the population does not benefit from integrated primary health care programs for early diagnosis and has less access to effective health services. Furthermore, CVDs are one of the factors that most contribute to increased health care costs.^3,4^

CVDs may be considered complex diseases, as they are influenced by both genetic predisposition and lifestyle.^5^ Several habits, customs, practices, and behaviors comprise a healthy lifestyle, with the following standing out: balanced diet, consumption of an appropriate amount of water, 7 to 8 hours of rest per day, and practice of physical activity.^6^ Some studies indicate that the higher the number of healthy habits followed, the lower the occurrence of cardiovascular conditions, the lower the mortality, and the greater the life expectancy.^5,7^

Some of the most important factors for heart diseases include inadequate diets, sedentary lifestyle, use of tobacco, and harmful use of alcohol. Furthermore, there are behavioral risk factors, which may be manifested through high blood pressure (BP), high blood glucose, hyperlipidemia, overweight, and obesity; additionally, these risk factors may indicate higher risk for the development of heart attacks, strokes, heart failure, and other complications.

Although several studies assess life habits of different professional categories in Brazil,^8,9^ the literature lacks data on the life habits of pharmaceutical industry professionals, an industry that since 2000 has been experiencing a significant growth in sales, volume of production, and size of companies.^10^

In light of the foregoing, the aim of this study was to elucidate the lifestyle of a sample of professionals from a pharmaceutical company located in the state of São Paulo, Brazil, and to evaluate cardiovascular risk factors.

Based on the results obtained, it will be possible to establish efficient internal policies for prevention and early diagnosis of CVDs, in order to reduce their impact on government health expenditure and increase workers’ quality of life.

## METHODS

The study was conducted in compliance with ethical standards and based on the ICH Harmonised Tripartite Guideline - E6 - Good Clinical Practice: Consolidated Guideline, on the Document of the Americas, and on the principles of the Declaration of Helsinki and its later amendments.

This research was submitted to Plataforma Brasil and was approved by the Research Ethics Committee under opinion number 3.811.884, with the title “Let’s know your health better? Assessment of lifestyle, BP, and cholesterol in the population of employes of Libbs Farmacêutica LTDA.”

Before starting the study, all participants received a detailed explanation about the procedures, objectives, benefits, and risks associated with the study; subsequently, the free consent form (ICF) was provided in copies, and possible questions were clarified.

A total of 1,875 employees of Libbs Farmacêutica LTDA participated in the study. Inclusion criteria were individuals of both sexes aged 20 years or older who accepted to participate in the study after reading and signing the ICF.

This was an observational, longitudinal, prospective study conducted from October 2019 to September 2020 that used a printed questionnaire to obtain information on the lifestyle and on parameters considered important for the detection of CVDs, such as BP, glycated hemoglobin, and cholesterol, from employees at a pharmaceutical company located in the state of São Paulo, Brazil.

In addition to the questionnaire, body mass index (BMI) was calculated, BP was measured using a digital oscillometric device (automatic blood pressure monitor, HEM 7200 model, Omrom Healthcare Co., Ltd., São Paulo, Brazil), and blood samples were collected to measure serum levels of total cholesterol (TC) and glycated hemoglobin. After puncture, the test was conducted in accordance with manufacturer’s instructions (Accutrend Plus system, F. Hoffman - La Roche Ltd, Basel, Switzerland). TC levels were measured in milligrams by deciliter (mg/dL).

The research was conducted during several internal institutional events and in punctual actions performed in the municipality of Embu das Artes (production unit). The sample was composed of employees working in different positions at the pharmaceutic company, such as production line workers and those engaged in the commercial, executive, and administrative functions. In all the scenarios, there was a space specifically assigned for the study.

Statistical analysis was performed using the SAS^®^ software, version 9.4. Data were graphically represented using descriptive measures such as mean, standard deviation, median, minimum and maximum values, and absolute and relative frequency. The Pearson’s chi-square test was used for correlation analyses.

## RESULTS

The analysis was performed based on data obtained from 1,875 participants in the study. The following correlations were evaluated: schooling and sex, smoking and sex; physical activity and sex; diet and sex; BMI and sex; TC and sex; glycated hemoglobin and sex; systolic BP (SBP) and sex; diastolic BP (DBP) and sex. Table 1 shows participants’ characteristics.

**Table 1:** Demographic data of the study population

	Sex				Total	
	Female		Male			
	(n = 856)		(n = 1,019)		(n = 1,875)	
	n	%	n	%	n	%
Schooling						
Complete/incomplete elementary education	20	2.3	20	2.0	40	2.1
Comple/incomplete high school education	133	15.5	255	25.2	388	20.8
Incomplete higher education	69	8.1	152	15.0	221	11.8
Complete higher education	343	40.1	353	34.9	696	37.3
Graduate education (MBA, specialization)	291	34.0	232	22.9	523	28.0
Total answers	856	100.0	1,012	100.0	1,868	100.0
Smoking						
Never smoked	725	84.9	826	81.5	1.551	83.0
Current smoker	36	4.2	54	5.3	90	4.8
Former smoker who quit smoking more than 5 years ago	55	6.4	78	7.7	133	7.1
Former smoker who quit smoking less than 1 year ago	23	2.7	35	3.5	58	3.1
Former smoker who quit smoking less than 5 years ago	15	1.8	21	2.1	36	1.9
Total answers	854	100.0	1,014	100.0	1,868	100.0
Physical activity						
I do not perform regular physical activity	446	52.1	453	44.7	899	48.1
I perform mild/moderate physical activity up to 3-4 times/week (less than 150 min)	331	38.7	405	39.9	736	39.4
I perform moderate/intense physical activity at least 5 times/week (more than 150 min)	79	9.2	156	15.4	235	12.6
Total answers	856	100.0	1,014	100.0	1,870	100.0
Consumption of fruits and vegetables						
Less than 2 servings per day	428	50.0	645	64.0	1.073	57.6
From 2 to 3 servings per day	359	41.9	306	30.4	665	35.7
4 or more servings per day	69	8.1	57	5.7	126	6.8
Total answers	856	100.0	1,008	100.0	1,864	100.0
Consumption of fish						
Do not consume	1	0.1	0	0.0	1	0.1
Less than once per week	538	63.0	649	64.5	1.187	63.8
Once or twice per week	268	31.4	304	30.2	572	30.8
More than twice per week	47	5.5	53	5.3	100	5.4
Total answers	854	100.0	1,006	100.0	1,860	100.0
Consumption of sugary drinks						
Less than 1 glass per day	500	58.5	457	45.7	957	51.6
From 1 to 2 glasses per day	237	27.7	345	34.5	582	31.4
More than 2 glasses per day	117	13.7	199	19.9	316	17.0
None of the above	1	0.1	0	0.0	1	0.1
Total answers	855	100.0	1,001	100.0	1,856	100.0
BMI range (kg/m^2^)						
Below 18.5	9	1.1	3	0.3	12	0.6
18.5-24.9	393	46.0	210	20.7	603	32.3
25.0-29.9	318	37.2	497	49.0	815	43.6
30.0-34.9	107	12.5	229	22.6	336	18.0
35.0-39.9	23	2.7	62	6.1	85	4.6
Equal to or above 40	4	0.5	13	1.3	17	0.9
Total answers	854	100.0	1,014	100.0	1,868	100.0
BMI (kg/m^2^)						
Mean ± SD	26 ± 4.1		28.4 ± 4.2		27.3 ± 4.3	
Median	25.3		27.8		26.8	
Minimum-maximum	11 ± 42		17.1 ± 44.7		11 ± 44.7	
Total answers	854		1,014		1,868	
Cholesterol range (mg/dL)						
Below 200	610	72.8	771	76.9	1,381	75.1
200-239	169	20.2	163	16.3	332	18.0
Equal to or above 240	59	7.0	68	6.8	127	6.9
Total answers	838	100.0	1,002	100.0	1,840	100.0
Cholesterol (mg/dL)						
Mean ± SD	179.6 ± 39.3		170.9 ± 41.7		174.8 ± 40.8	
Median	177.5		166.0		171.0	
Minimum-maximum	90 ± 352		90 ± 334		90 ± 352	
Total answers	838		1,002		1,840	
Glycated hemoglobin range (%)						
Below 5.7	747	88.1	849	84.2	1.596	86.0
From 5.7 to 6.4	89	10.5	138	13.7	227	12.2
Equal to or above 6.5	12	1.4	21	2.1	33	1.8
Total answers	848	100.0	1,008	100.0	1,856	100.0
Glycated hemoglobin (%)						
Mean ± SD	5.1 ± 0.5		5.3 ± 0.6		5.2 ± 0.6	
Median	5.1		5.2		5.2	
Minimum-maximum	4 ± 8.5		4 ± 11.6		4 ± 11.6	
Total answers	847		1,008		1,855	
SBP range (mmHg)						
Below 120	398	46.5	170	16.7	568	30.3
From 120 to 139	364	42.6	527	51.8	891	47.6
From 140 to 159	75	8.8	278	27.3	353	18.8
Equal to or above 160	18	2.1	43	4.2	61	3.3
Total answers	855	100.0	1,018	100.0	1,873	100.0
SBP (mmHg)						
Mean ± SD	121.9 ± 15		133.3 ± 14.7		128.1 ± 15.9	
Median	120.0		132.0		127.0	
Minimum-maximum	11 ± 187		93 ± 214		11 ± 214	
Total answers	855		1,018		1,873	
DBP range (mmHg)						
Below 80	575	67.3	475	46.7	1.050	56.1
From 80 to 89	187	21.9	334	32.8	521	27.8
From 90 to 99	71	8.3	170	16.7	241	12.9
Equal to or above 100	22	2.6	38	3.7	60	3.2
Total answers	855	100.0	1,017	100.0	1,872	100.0
DBP (mmHg)						
Mean ± SD	76.5 ± 10		80.7 ± 12.1		78.8 ± 11.4	
Median	75.0		80.0		78.0	
Minimum-maximum	39 ± 121		10 ± 132		10 ± 132	
Total answers	855		1,017		1,872	

BMI = body mass index; DBP = diastolic blood pressure; MBA = master in business administration; SBP = systolic blood pressure; SD = standard deviation.

### HABITS: SMOKING, PHYSICAL ACTIVITY, AND DIET

Of the respondents, 54.3% were male, 65.3% had at least complete higher education, 83% had never smoked, and 48.1% did not perform regular physical activity. With regard to diet, 57.6% consumed at least two servings of fruits and vegetables per day, 63.8% consumed fish less than once per week, and 51.6% consumed less than one glass of sugary drinks per day.

Statistically significant differences were found between the genders (p < 0.001): higher education or graduate education was 16.3% more frequent in women, not performing physical activity was 7.4% more common in women, and performing physical activity more times per week was 6.2% more common in men; furthermore, women consume less sugary drinks and more fruits and vegetables than men.

**Figure 1:**
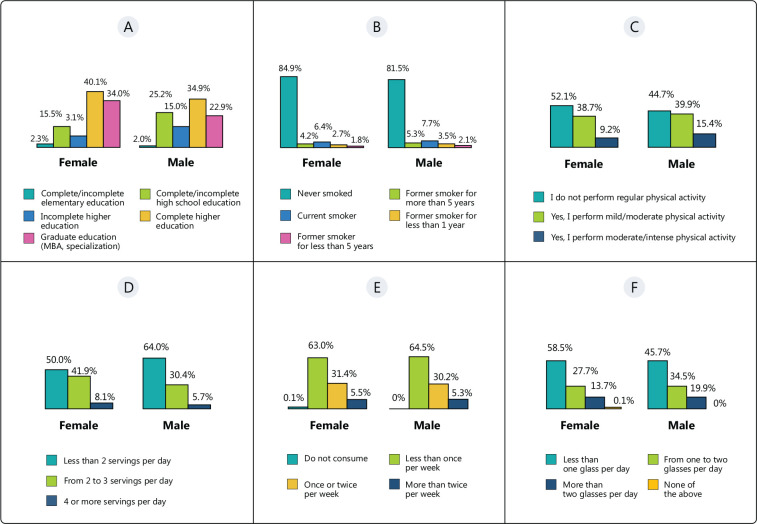
Correlation between habits and sex. Schooling (A), smoking (B), physical activity (C), consumption of fruits and vegetables (D), consumption of fish (E), and consumption of sugary drinks (F).

The correlation between life habits and sex is shown in Figure 1.

### BODY MASS INDEX

**Figure 2:**
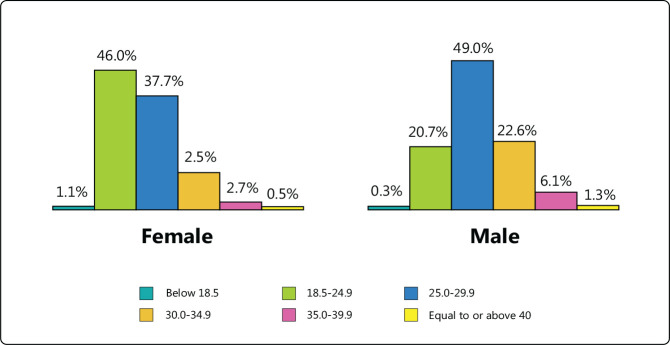
Correlation between body mass index and sex.

Most respondents had a BMI from 25 to 29.9 m/kg^2^ (43.6%) or from 18.5 to 24.9 m/kg^2^ (32.3%). Overall data are presented in Figure 2, showing the relationship between BMI and sex. BMI below 25 m/kg was 26.1% more frequent in women (p < 0.001).

### COMPLEMENTARY TESTS

**Figure 3:**
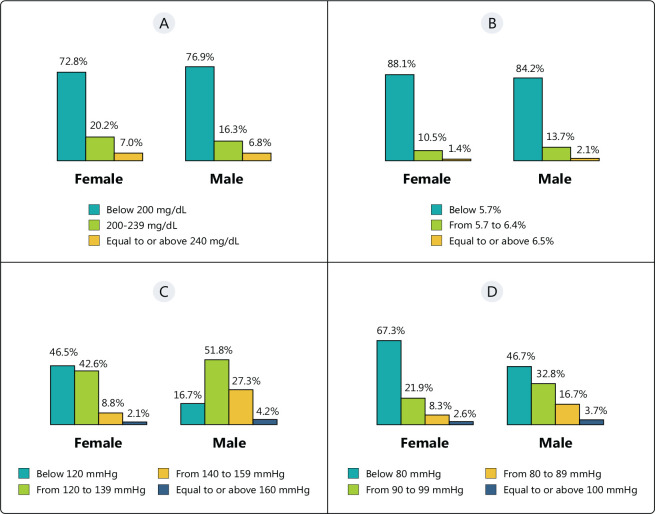
Correlation between test and sex. Total cholesterol (A), glycated hemoglobin (B), systolic blood pressure (C), and diastolic blood pressure (D).

Most respondents had TC levels below 200 mg/dL (75.1%) and glycated hemoglobin below 5.7% (86.0%); furthermore, a great number had a SBP from 120 to 139 mmHg (47.6%) and a DBP below 80 mmHg (56.1%). BP measurements were significantly lower in women (p < 0.001): 30% more women had a SBP below 120 mmHg, and 20.6% more women had a DBP below 80 mmHg. The correlation between the data obtained and participants’ sex is presented in Figure 3.

## DISCUSSION

In a study assessing health habits in the population of the city of São Paulo, Brazil, the prevalence of health lifestyle was 36.9% in the elderly, 15.4% in adults, and 9.8% in adolescents, and was higher in females in the elderly and adults. Healthy lifestyle was defined on the basis of physical activity, diet, smoking, and alcohol abuse and addiction, according to the respective guidelines. Among individuals with unhealthy lifestyle, 32.2% of adults failed to reach the guidelines for adequate diet.^11^

In the abovementioned study, food consumption was measured by the Healthy Eating Index, which consists of 10 dietary components and ranges from 0 to 100 points. The higher the score, the better the quality of life. Participants were categorized into terciles, according to their Healthy Eating Index, and those in the third tercile were classified as having an adequate diet. Complementarily, food consumption was the main factor associated with unhealthy lifestyle^11^.

According to the characteristics of the sample assessed in the present study, 83% of participants had never smoked, with no great differences between men and women. Although smoking is still an important risk for CVDs and the leading cause of preventable death worldwide,^12^ its control in Brazil is one of the most successful cases of a public health policy in the world.^13^ Recent investigations conducted by the Brazilian Health Ministry revealed that Porto Alegre, with 14.4% of prevalence of smoking, São Paulo, with 12.5%, Curitiba, with 11.4%, and Florianópolis, with 11.2%, were the state capitals with the highest number of smokers.^14^

Nearly half the sample did not perform regular physical activity. According with data from the World Health Organization, the percentage of adults classified as physically inactive in Brazil was 47%,^15^ a percentage close to that found in this study, which revealed that 48.1% of the sample did not perform regular physical activity.

According to data published in 2020 by the Brazilian Institute of Geography and Statistics (Instituto Brasileiro de Geografia e Estatística, IBGE) comparing the frequency of food consumption according to sex, men consumed fewer vegetables, greens, and fruits, and women consumed more cookies, cakes, sweets, milk, dairies, coffee, and tea.^16^ In turn, more than a half of participants in this study (both men and women) consumed less than two servings of fruits and vegetables per day (57.6%). With regard to fish consumption, although it was not analyzed with the same parameters used in the present study, the IBGE found that the rural population is the greatest consumer of fish. Fresh fish and fish-based foods are more consumed by lower-income people than by higher-income people.^16^

With regard to BMI, the prevalence of overweight and obesity in Brazil continuously increased from 1974-1975 to 2008-2009 for both sexes. Compared with more recent research, this trend was maintained, with the prevalence of overweight increasing from 49% in 2008-2009 to 57% in 2013, and that of obesity increasing from 15% to 21% in the same period.^17^ In the sample analyzed in the present study, although there was a difference between men and women, no important differences were observed when data were analyzed as a whole. It was found that 43.6% of the sample had overweight, and 23.5% had different levels of obesity, which an extremely relevant factor for cardiovascular risk.

According to a recent investigation, 23.2% of the world’s adult population in 2005 was overweight (24.0% in men [23.4-24.5%] and 22.4% in women [21.9-22.9%]) and 9.8% (9.6-10.0%) was obese (7.7% in men [7.4-7.9%] and 11.9% in women [11.6-12.2%]). By 2030, the respective number of overweight and adults was projected to be 1.35 billion and 573 million individuals without adjusting for secular trends. If recent secular trends continue unabated, the absolute numbers were projected to total 2.16 billion overweight and 1.12 billion obese individuals.^18^

The relationship between sex and prevalence of dyslipidemia is not well established in the literature. Nonetheless, evidence shows a higher prevalence of dyslipidemia in women.^19^ According to a descriptive study using laboratory data from the Brazilian National Health Survey (NHS) collected between 2014 and 2015, the prevalence of TC ≥ 200 mg/dL in the population was 32.7%, being higher in women (35.1%).^20^ This percentage is very close to that obtained in the present study, since 24.9% of participants had TC levels above 200 mg/dL, although no difference was observed in terms of sex.

A study presenting data from the Brazilian NHS found that 6.6% of adults have glycated hemoglobin ≥ 6.5%; and the proportion of intermediate hyperglycemia, or pre-diabetes, was 6.8% when defined by the criteria of the International Expert Committee. Across all the criteria, the prevalence was highest among women.^21^ However, in the present study the percentage of participants with glycated hemoglobin ≥6.5% was much lower, accounting for 1.4 and 2.1% for women and men, respectively.

According to the Brazilian Guidelines of Arterial Hypertension (AH), stage I AH occurs when SBP is between 140-159 mmHg and DBP between 90-99 mmHg. The prevalence of this disease varies based on study methods and subjects. According to the 2013 NHS, 21.4% (95% CI 20.8-22.0) of Brazilian adults self-reported as having AH, with a higher prevalence among men.^22^ In the population studied in this observational research, findings are line with those of the literature, with men showing a higher percentage of hypertension (31.5%) compared with women (10.9%), and a mean between sexes of 22.1%.

The limitations identified in this study include the fact that the population does not reflect the entire context of the pharmaceutical industry, since each company can implement programs to improve the quality of life of its employees. Furthermore, the instrument used requires the respondents to provide truthful answers, especially in those related to dietary habits, because each individual can possibly make a subjective analysis when answering the questionnaire.

## CONCLUSIONS

The results of this study are consistent with those described in the literature for the Brazilian population, which indicate the possibility of improving healthy habits, such as quit smoking, balanced diet, and regular physical activity, a key factor to reduce cardiovascular risk.

Actions aiming to evaluate the life habits of workers at company, such as that conducted in the present study, may have an impact on employer’s cost on employee’s health, in addition to being considered a social responsibility action.

The improvement of these habits, with the help of efficient public policies that promote positive changes in parameters such as BMI, TC, glycated hemoglobin, and BP. Thereby, it is possible to improve prevention and early diagnosis of CVDs, so as to reduce the impact of government health expenses.
